# Invasive pneumococcal disease surveillance in Canada, 2021–2022

**DOI:** 10.14745/ccdr.v50i05a02

**Published:** 2024-05-24

**Authors:** Averil Griffith, Alyssa R Golden, Brigitte Lefebvre, Allison McGeer, Gregory J Tyrrell, George G Zhanel, Julianne V Kus, Linda Hoang, Jessica Minion, Paul Van Caeseele, Hanan Smadi, David Haldane, Yang Yu, Xiaofeng Ding, Laura Steven, Jan McFadzen, Kristyn Franklin, Irene Martin

**Affiliations:** 1 National Microbiology Laboratory, Public Health Agency of Canada, Winnipeg, MB; 2 Laboratoire de santé publique du Québec, Sainte-Anne-de- Bellevue, QC; 3 Toronto Invasive Bacterial Diseases Network (TIBDN), Department of Microbiology, Mount Sinai Hospital, Toronto, ON; 4 Provincial Laboratory for Public Health, Edmonton, AB; 5 Department of Medical Microbiology and Infectious Diseases, Max Rady College of Medicine, Rady Faculty of Health Sciences, University of Manitoba, Winnipeg, MB; 6 Public Health Ontario, Toronto, ON; 7 Department of Laboratory Medicine and Pathobiology, University of Toronto, Toronto, ON; 8 British Columbia Centre for Disease Control, Vancouver, BC; 9 Roy Romanow Provincial Laboratory, Regina, SK; 10 Cadham Provincial Laboratory, Winnipeg, MB; 11 New Brunswick Department of Health, Fredericton, NB; 12 Queen Elizabeth II Health Science Centre, Halifax, NS; 13 Newfoundland and Labrador Public Health Laboratory, St. John’s, NL; 14 Queen Elizabeth Hospital, Charlottetown, PE; 15 Stanton Territorial Hospital Laboratory, Yellowknife, NT; 16 Yukon Communicable Disease Control, Whitehorse, YT; 17 Centre for Emerging and Respiratory Infections and Pandemic Preparedness, Public Health Agency of Canada, Ottawa, ON

**Keywords:** invasive pneumococcal disease, IPD, Canada, *Streptococcus pneumoniae*, PCV13, pneumococcus, serotype, surveillance, antimicrobial resistance

## Abstract

**Background:**

Invasive pneumococcal disease (IPD, *Streptococcus pneumoniae*) has been a nationally notifiable disease in Canada since 2000. The use of conjugate vaccines has caused a shift in the distribution of serotypes over time. This report is a summary of the demographics, serotypes and antimicrobial resistance of IPD isolates collected in Canada in 2021 and 2022.

**Methods:**

The National Microbiology Laboratory (NML) of the Public Health Agency of Canada in Winnipeg, Manitoba collaborates with provincial and territorial public health laboratories to conduct national surveillance of IPD. There were 1,999 isolates reported in 2021 and 3,775 isolates in 2022. Serotype was determined by the Quellung reaction or whole-genome sequencing (WGS). Antimicrobial susceptibilities were determined by WGS methods, broth microdilution, or data shared by collaborators in the Canadian Antimicrobial Resistance Alliance program at the University of Manitoba. Population-based IPD incidence rates were obtained through the Canadian Notifiable Disease Surveillance System.

**Results:**

The incidence of IPD in Canada was 5.62 cases per 100,000 population in 2021, decreasing from the peak of 10.86 cases per 100,000 population in 2018. Serotypes with increasing trends (*p*<0.05) between 2018 and 2022 included: 4 (6.1%–12.4%), 9V (1.0%–5.1%) and 12F (4.8%–5.4%). The overall prevalence of PCV13 serotypes increased over the same period (31.2%−41.5%, *p*<0.05) while the prevalence of non-vaccine types decreased significantly (27.3%–21.5%, *p*<0.0001). The highest rates of antimicrobial resistance in 2021 and 2022 were seen with clarithromycin (21%, 2021; 24%, 2022) and erythromycin (22%, 2021; 24%, 2022). Multidrug-resistant IPD continued to increase from 2018 to 2022 (6.7%–12.6%, *p*<0.05).

**Conclusion:**

The number of cases of IPD continued to decrease in 2021 in comparison to previous years, however, 2022 saw a return to pre-COVID-19 levels. Disease due to PCV13 serotypes 3, 4, 9V and 19F, as well as non-PCV13 serotypes 12F and 20, is increasing in prevalence. Surveillance of IPD to monitor changing serotype distribution and antimicrobial resistance is essential.

## Introduction

*Streptococcus pneumoniae*, the causative agent of invasive pneumococcal disease (IPD), is responsible for severe infections worldwide, such as meningitis and bacteremia, with children, the elderly and immunocompromised individuals being at greatest risk (1). The majority of cases can be attributed to a small subset of serotypes despite there being over 100 distinct types; vaccination strategies have been successful in reducing the incidence of these types (1,2). Pneumococcal conjugate vaccines (PCV), PCV7 (containing serotypes 4, 6B, 9V, 14, 18C, 19F, and 23F), PCV10 (PCV7 serotypes plus 1,5 and 7F), and PCV13 (PCV10 serotypes plus 3,6A and 19A) were introduced in Canada between 2002 and 2011 (3–7). These vaccines have been successful in decreasing the incidence of their constituent serotypes, however, subsequent increases in non-vaccine serotypes continue (3,4,8). PPV23, a 23-valent pneumococcal polysaccharide vaccine (which includes all PCV13 serotypes except 6A, plus serotypes 2, 8, 9N, 10A, 11A, 12F, 15B/C, 17F, 20, 22F and 33F) has been available for use in Canada since 1989 for adults and people over two years of age at high risk of IPD (6,9).

In 2023, the National Advisory Committee on Immunization (NACI) recommended the use of a 15-valent vaccine (PCV15: PCV13 serotypes plus 22F and 33F) for all ages older than six weeks (10,11). A 20-valent vaccine (PCV20: PCV15 serotypes plus 8, 10A, 11A, 12F and 15B/C) has been recommended for use in seniors over 65 years and for adults between 18 and 64 years with underlying medical conditions (12,13).

The objective of this annual surveillance report is to provide a summary of the serotypes and antimicrobial resistance associated with IPD in Canada in 2021 and 2022.

## Methods

### Surveillance program

Canadian surveillance of IPD consists of a passive laboratory-based system where invasive isolates from the provincial and territorial public health laboratories are sent to either the National Microbiology Laboratory (NML) in Winnipeg, Manitoba, the Alberta Public Health Laboratory (ProvLab), or the *Laboratoire de santé publique du Québec* (LSPQ) for serotyping. There were 1,999 IPD isolates reported in 2021 and 3,775 isolates reported in 2022 (**Table 1** and **Table 2**), including isolates serotyped by LSPQ (n=353, 2021; n=708, 2022) and ProvLab (n=302, 2021; n=643, 2022). An expansion of IPD surveillance in Québec occurred in 2019 to include all invasive strains. Sterile clinical isolation sites include blood, cerebrospinal fluid, peritoneal, pericardial or joint fluid, internal body sites, and deep tissue, including surgical or biopsy samples. For this report, isolates from pleural fluid (empyema) are included, despite not meeting the current national case definition for invasive disease, as they are widely considered as invasive in other jurisdictions (3).

**Table 1:** Number of invasive *Streptococcus pneumoniae* isolates submitted by province, 2021

Province	Age group (years)						Not given	Total
	<2	2–4	5–14	15–49	50–64	≥65		
British Columbia^a^	4	4	5	90	93	82	0	278
Alberta	4	9	3	131	87	65	3	302
Saskatchewan	2	4	1	45	28	24	0	104
Manitoba	9	8	3	58	38	25	0	141
Ontario	51	22	13	153	162	204	5	610
Québec	45	23	8	90	120	165	0	451
Atlantic^b^	4	1	0	17	33	30	3	88
Northern^c^	0	1	0	11	13	0	0	25
**Total**	**119** **(6%)**	**72** **(4%)**	**33** **(2%)**	**595** **(30%)**	**574** **(29%)**	**595** **(30%)**	**11** **(1%)**	**1,999**

^a^ Includes isolates from Yukon

^b^ Includes isolates from New Brunswick, Prince Edward Island, Nova Scotia, and Newfoundland and Labrador

^c^ Includes isolates from the Northwest Territories and Nunavut

Note: Population-based incidence of disease data for 2009 to 2021 were obtained through the Canadian Notifiable Disease Surveillance System (CNDSS). Population data for incidence rates were obtained from Statistics Canada’s annual population estimates

**Table 2:** Number of invasive *Streptococcus pneumoniae* isolates submitted by province, 2022

Province	Age group (years)						Not given	Total
	<2	2–4	5–14	15–49	50–64	≥65		
British Columbia^a^	10	10	12	172	134	176	2	516
Alberta	22	15	17	272	177	135	2	640
Saskatchewan	8	9	5	109	60	47	0	238
Manitoba	10	2	8	97	61	55	0	233
Ontario	64	59	48	260	340	395	4	1,170
Québec	46	25	22	156	183	365	0	797
Atlantic^b^	6	3	10	26	44	65	5	159
Northern^c^	0	0	1	8	9	4	0	22
**Total**	**166** **(4%)**	**123** **(3%)**	**123** **(3%)**	**1,100** **(29%)**	**1,008** **(27%)**	**1,242** **(33%)**	**13** **(0.3%)**	**3,775**

^a^ Includes isolates from Yukon

^b^ Includes isolates from New Brunswick, Prince Edward Island, Nova Scotia, and Newfoundland and Labrador

^c^ Includes isolates from the Northwest Territories and Nunavut

### Isolate testing

Invasive pneumococcal disease isolates were screened using bile solubility and optochin disc susceptibility at NML until October 2022, when bile solubility was discontinued (Oxoid) (14). Serotyping of IPD at LSPQ and ProvLab Alberta was performed by the Quellung reaction using commercial antisera (SSI Diagnostica; Statens Serum Institut, Copenhagen, Denmark) (15). Serotyping at NML was performed by the Quellung reaction until October 2022; from November 2022 to December 2022, whole-genome sequencing (WGS) was carried out on all isolates submitted to NML using the Illumina platform, with serotypes identified directly using the WGS Analysis and Detection of Molecular Markers (WADE) pipeline, as described elsewhere (16). Isolates that were non-typeable by WGS were confirmed by the Quellung reaction and the National Center for Biotechnology Information (NCBI)’s Basic Local Alignment Search Tool (BLAST) analysis of the rpoB gene (15,17). For this study, serotypes 15B and 15C were grouped together as 15B/C because of reported reversible switching between them *in vivo* during infection, making it difficult to differentiate between the two types (18,19).

Antimicrobial susceptibility testing (AST) was performed on most 2021 IPD isolates submitted to NML for serotyping by the provincial public health laboratories (Saskatchewan, Manitoba, Ontario, Québec, Nova Scotia, Prince Edward Island, Newfoundland and Labrador, and six of eight health regions in New Brunswick). In collaboration with the University of Manitoba and the Canadian Antimicrobial Resistance Alliance, minimum inhibitory concentrations were determined using in-house broth microdilution in accordance with Clinical & Laboratory Standards Institute (CLSI) guidelines (20,21). Minimum inhibitory concentrations for 2022 isolates were determined using a combination of WGS-predicted susceptibility and in-house broth microdilution (20–22). Antimicrobials included in this report are penicillin, ceftriaxone, chloramphenicol, clarithromycin, clindamycin, doxycycline, erythromycin, trimethoprim/sulfamethoxazole, linezolid, and vancomycin. Minimum inhibitory concentration interpretive standards were defined according to CLSI breakpoints (21). Multidrug resistance (MDR) was defined as resistance to three or more classes of antimicrobials for this report.

### Data analysis

As previously described (23), data submitted with bacterial isolates included patient age, sex, clinical source, province, and date of collection. Duplicate isolates collected from the same patient within 14 days were counted once if they were the same serotype, with the most invasive isolation site assigned. Meningitis-related isolates were regarded as most invasive, followed by blood and then other sterile sites. Data was aggregated by age into <2, 2–4, 5–14, 15–49, 50–64, and ≥65-year-old age groups, and regionally into Western (British Columbia, Alberta, Saskatchewan, Manitoba), Central (Ontario, Québec), Eastern (New Brunswick, Nova Scotia, Prince Edward Island, Newfoundland and Labrador) and Northern (Yukon, Northwest Territories and Nunavut) regions of Canada. Statistical significance of trends was assessed using the Cochran-Armitage test of trend, with a *p*-value of <0.05 considered to be statistically significant.

## Results

**Figure 1:**
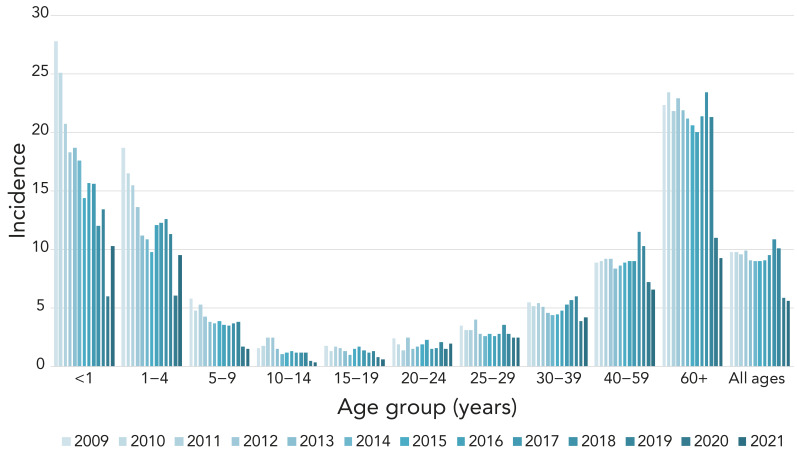
Annual incidence of invasive pneumococcal disease cases per 100,000 population in Canada by age group, 2009–2021^a^ ^a^ Data from Canadian Notifiable Disease Surveillance System (CNDSS); 2022 data not available at time of writing

Overall IPD incidence rates in Canada remained stable from 2009 to 2019 (9.8–10.1), after which there was a decline in 2020 and 2021 to fewer than six cases per 100,000 population (**Figure 1**, **Appendix, Supplemental Table S1**).

There was a large increase in the number of isolates submitted in 2022 (n=3,775) compared to 2021 (n=1,999), particularly in the first and last quarters of 2022 (Appendix, **Figure S1**). The distribution among age groups was consistent year-to-year. Infants <2 years of age accounted for 4%–6% of isolates, toddlers aged 2–4 years for 3%–4%, children aged 5 to 14 years for 2%–3%, patients aged 15 to 49 years for 29%–30%, older adults aged 50 to 64 years for 27%–29% and seniors aged ≥65 years for 30%–33% (Table 1 and Table 2). Of the isolates with gender information available, isolates from male patients represented 58.2% (n=1,152) and 57% (n=2,152) of isolates collected in 2021 and 2022, respectively. Blood was the most frequent clinical isolation site, accounting for 94% (n=1,877) of isolates in 2021 and 92% (n=3,460) in 2022. Additional information on specimen source by age and serotype are available in Appendix, **Figures S2 to S5**.

**Figure 2:**
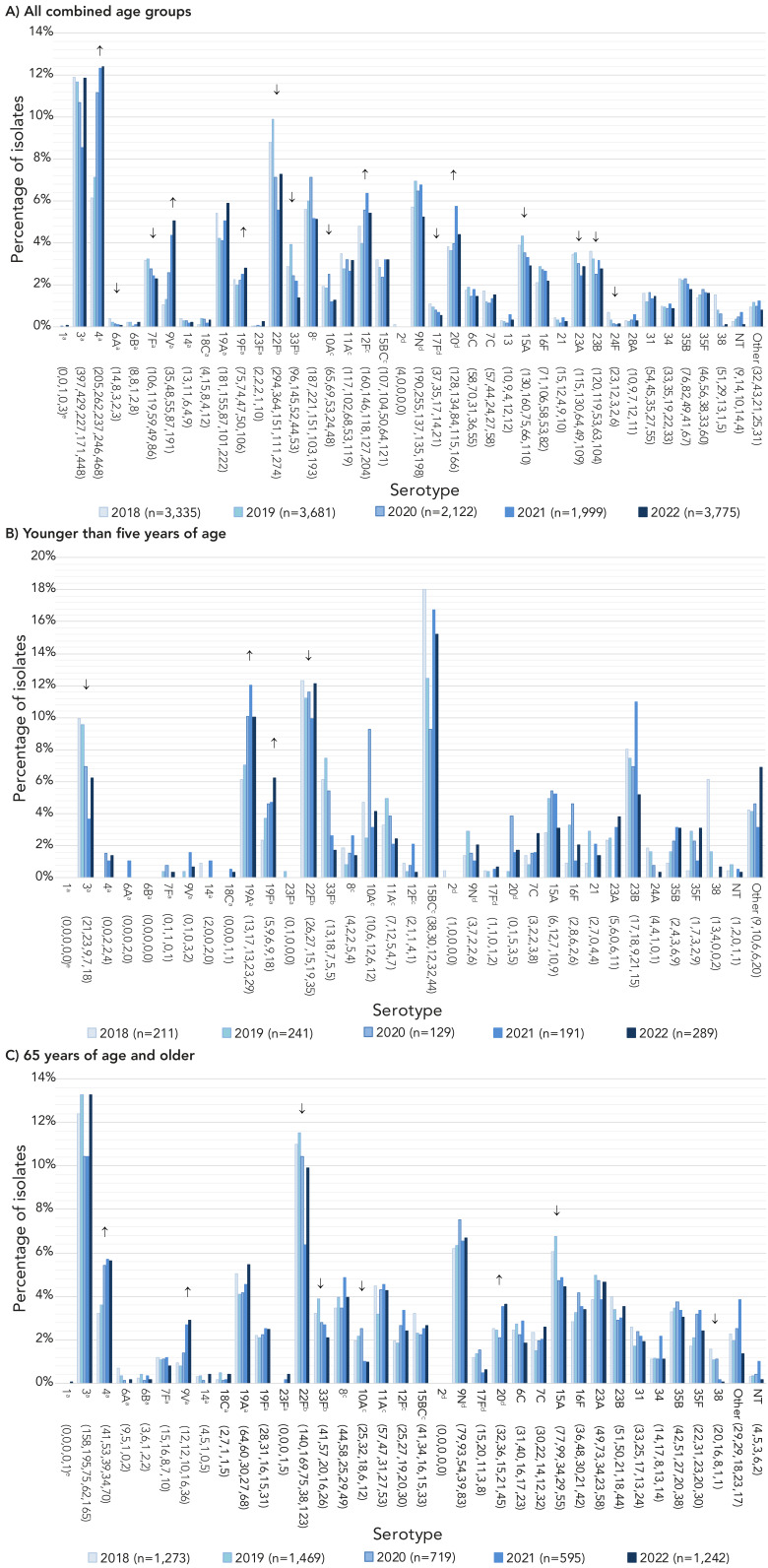
Invasive *Streptococcus pneumoniae* serotype prevalence trends by age, 2018–2022^a,b,c,d,e,f,g^ ^a^ Component of PCV13 ^b^ Component of PCV15 ^c^ Component of PCV20 ^d^ Component of PPV23 ^e^ Number of isolates for 2018, 2019, 2020, 2021 and 2022, respectively ^f^ For serotypes with an overall (2018–2022) N≥30: up or down arrows indicate statistically significant trends toward increasing or decreasing prevalence for the 2018–2022 timespan, using the chi-squared test for trend. Serotypes with no arrow either did not demonstrate a statistically significant trend, or did not have an overall N≥30 ^g^ Serotypes 15B and 15C were grouped together as 15B/C because of reported reversible switching between them *in vivo* during infection, making it difficult to precisely differentiate between the two types (18,19). Trends for more detailed age groups can be found in the Appendix, Figures S8 to S12

The most commonly collected serotypes overall in both 2021 and 2022 were 4 (12.3%, n=246 and 12.4%, n=468) and 3 (8.6%, n=171 and 11.9%, n=448) (**Figure 2**, A). Other common serotypes included 22F, 19A, 12F and 9N. Serotypes that demonstrated significant increasing trends in prevalence from 2018 to 2022 include PCV13 serotypes 4 (6.1%−12.4%, *p*<0.001), 9V (1.0%−5.1%, *p*=0.011) and 19F (2.2%−2.8%, *p*=0.0422), as well as 12F (4.8%–5.4%, *p*=0.0068) and 20 (3.8%–4.4%, *p*=0.0143) (Figure 2A). Vaccine serotypes that significantly decreased in prevalence from 2018 to 2022 include 22F, 33F (*p*<0.0001) and 6A, 7F, 10A and 17F (*p*≤0.007) (Figure 2A).

The three most common serotypes in children <2 years during 2021 and 2022, respectively, included 15B/C (10.9%, 15.1%), 22F (12.6%, 10.8%), and 19A (12.6%, 9.6%), while the most common for 2 to 4-year-olds was serotype 15B/C (26.4%, 15.4%), followed by 22F (5.6%, 13.8%). Serotypes 22F (18.2%, 17.1%), 3 (3.0%, 12.2%) and 19F (15.2%, 11.4%) were the most common in 5 to 14-year-olds. Serotype 4 was the most prevalent serotype in 15 to 49-year-olds (22.7%, 21.8%) followed by serotypes 12F (9.1%, 10.5%) and 3 (7.4%, 9.3%). Serotypes 4 (12.7%, 14.9%) and 3 (9.9%, 14.6%) were the most common in 50 to 64-year-olds, while serotypes 3 (10.4%, 13.3%) and 22F (6.4%, 9.9%) were dominant in adults over 65 years of age. See Figure 2 and Appendix, **Figures S6 to S7**.

Significant increases of serotypes 19A (7.1%–10.0%, *p*=0.04) and 19F (3.7%–6.2%, *p*=0.035) were observed in children <5 years of age from 2018 to 2022 (Figure 2B). Serotype 19F also increased significantly for children 5 to 14 years (4.4%–11.4%, *p*=0.0265). Patients 15 to 49 years of age saw significant increases in serotypes 4 (11.7%–21.8%, *p*<0.0267) and 9V (1.4%–7.6%, *p*<0.0001). Adults 50 to 64 years of age saw similar increases in serotypes 4 (7.8%–14.9%, *p*<0.0001) and 9V (1.4%–6.1%, *p*<0.0001). Significant increases for seniors ≥65 years were noted for serotype 4 (3.2%−5.6%, *p*=0.0003), 9V (0.9%–2.9%, *p*<0.0001) and 20 (2.5%–3.6%, *p*=0.0039) (Figure 2C). Serotypes 6A, 7F, 22F, 33F, 10A, 17F, 15A 23A, 23B, 24F and 38 all showed significant decreases from 2018 to 2022 for all combined age groups (*p*≤0.047) (Figure 2A).

Regionally, the top two serotypes associated with Western Canada, 4 (18.2%, 2021; 18.0%, 2022) and 3 (8.0%, 2021; 11.5%, 2022), remained the same as in previous years. In Central Canada, serotype 3 continued to be the most prevalent (9.1%, 2021; 12.1%, 2022), followed by 19A (7.4%, 2021; 8.4%, 2022). In Eastern Canada, serotypes 20 (15.9%) and 22F (11.4%) were the most common in 2021, while serotypes 4 (15.7%) and 3 (14.5%) were predominant in 2022. Serotype 4 continues to dominate in Northern Canada (81%, 2021; 34%, 2022) (Appendix, **Figures S13 to S17**).

**Figure 3:**
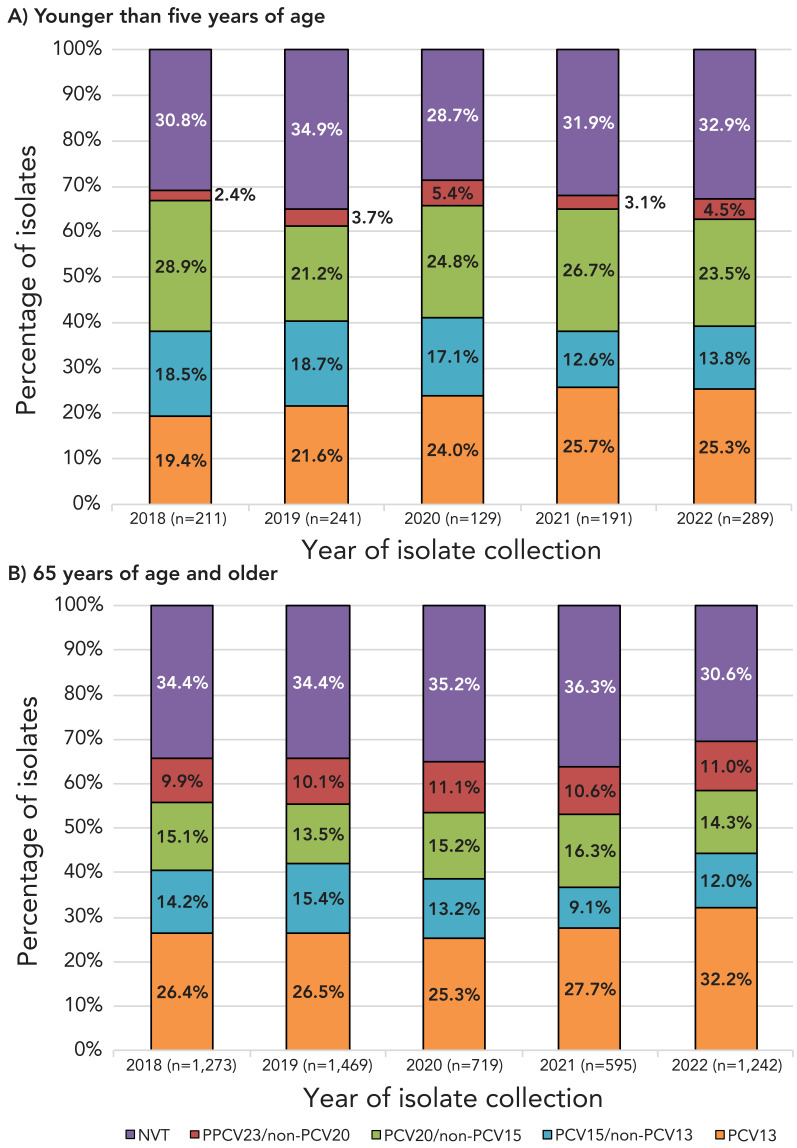
Invasive *Streptococcus pneumoniae* serotype trends by vaccine and age^a^, 2018–2022 Abbreviations: NVT, non-vaccine serotype; PCV, pneumococcal conjugate vaccine; PPCV, pneumococcal polysaccharide vaccine ^a^ Vaccine serotypes include PCV13 (1, 3, 4, 5, 6A/C, 6B, 7F, 9V, 14, 19A, 19F, 18C, 23F); PCV15 (all PCV13 serotypes plus 22F and 33F); PCV20 (all PCV15 serotypes plus 8, 10A, 11A, 12F, 15B/C) and PPV23 (PCV20 serotypes except 6A, plus 2, 9N, 17F, 20); NVT=all serotypes not included in PCV13, PCV15, PCV20 and PPV23. Trends for more detailed age groups can be found in the Appendix, Figures S18 to S23 and Tables S2 to S8

Serotypes belonging to the currently recommended PCV13 vaccine have significantly increased in prevalence overall from 2018 to 2022 (31.2%−41.5%, *p*=0.0269); this increase was seen in all age groups except children from 2 to 14 years. The proportion of PCV15-unique serotypes decreased significantly overall (11.7%–8.7%, *p*<0.0001), however, there was no significant change in the under 15-year age group. Proportions of PCV20-unique and PPV23-unique serotypes have not significantly changed from 2018 to 2022 among the age groups. The number of non-vaccine serotypes overall has decreased from 2018 to 2022 (27.3%–21.5%, *p*<0.001) (**Figure 3**, Appendix, Figures S18 to S23 and Tables S2 to S8).

Due to the different AST methods used for 2021 and 2022, the total number of isolates tested for each antimicrobial varied. The highest rate of resistance for both 2021 and 2022 was for clarithromycin (20.9%, 2021; 24.1%, 2022) (**Table 3**). Penicillin resistance increased significantly from 3.4% in 2018 to 8.3% in 2022 (*p*<0.0001), as did doxycycline resistance (8.5%–17.15%, *p*<0.0001) and trimethoprim-sulfamethoxazole resistance (7.5%–14.9%, *p*<0.0001). Significant decreases were seen for chloramphenicol resistance (5.4%–2.7%, *p*=0.01) and erythromycin resistance (25.8%–24.0%, *p*<0.0001). Resistance to ceftriaxone remains low, ranging from a high of 1.0% to a low of 0.3% between 2018 and 2022 (Table 3). All isolates were susceptible to linezolid and vancomycin. Resistance rates for specific serotypes are listed in **Table 4** and **Table 5**.

**Table 3:** Proportion of antimicrobial resistant invasive *Streptococcus pneumoniae* isolates by year, 2018–2022

Antimicrobial	Year (n, %)				
	2018	2019	2020	2021	2022
AXO	12 (0.7%)	6 (0.3%)	3 (0.3%)	10 (1.0%)	4 (0.4%)
CHL	100 (5.4%)	59 (3.2%)	43 (4.0%)	32 (3.2%)	29 (2.7%)
CLA	465 (26.2%)	473 (26.1%)	243 (23.7%)	195 (20.9%)	249 (24.1%)
CLI	128 (6.9%)	166 (8.9%)	86 (8.0%)	79 (8.0%)	88 (8.1%)
DOX	152 (8.5%)	216 (11.9%)	126 (12.2%)	135 (14.5%)	177 (17.2%)
ERY	31 (25.8%)	75 (43.9%)	54 (44.3%)	110 (21.6%)	260 (24.0%)
LEV	5 (0.3%)	9 (0.5%)	1 (0.1%)	0 (0.0%)	2 (0.2%)
PEN	63 (3.4%)	48 (2.6%)	36 (3.4%)	46 (4.7%)	90 (8.3%)
SXT	139 (7.5%)	177 (9.5%)	117 (11.0%)	105 (10.6%)	161 (14.9%)

Abbreviations: AXO, ceftriaxone using the parenteral meningitis Clinical and Laboratory Standards Institute interpretive standard; CHL, chloramphenicol; CLA, clarithromycin; CLI, clindamycin; DOX, doxycycline; ERY, erythromycin; LEV, levofloxacin; PEN, penicillin using the parenteral meningitis Clinical and Laboratory Standards Institute interpretive standard; SXT, trimethoprim/sulfamethoxazole

**Table 4:** Percentage of antimicrobial resistance of invasive *Streptococcus pneumoniae* serotypes collected, 2021

Serotype	Percentage of isolates with antimicrobial resistance^a^							
	PEN	AXO	ERY	CLA	CLI	CHL	DOX	SXT
3^b^	-	-	5%	8%	6%	13%	14%	-
4^b^	-	-	4%	11%	9%	10%	17%	1%
6A^b^	-	-	100%	100%	-	-	-	50%
9V^b^	26%	11%	33%	29%	-	-	23%	29%
14^b^	-	-	100%	100%	67%	-	-	67%
18C^b^	-	-	100%	25%	-	-	25%	25%
19A^b^	35%	10%	82%	74%	42%	6%	44%	32%
19F^b^	11%	7%	20%	13%	15%	-	13%	7%
22F^c^	-	-	50%	56%	2%	-	2%	-
33F^c^	-	-	67%	77%	-	-	-	-
8^d^	-	-	-	-	-	-	-	2%
10A^d^	-	-	-	-	-	-	-	8%
11A^d^	-	-	24%	23%	-	-	-	19%
12F^d^	-	-	25%	20%	-	2%	69%	69%
15B/C^d,e^	-	-	42%	35%	10%	-	3%	3%
9N^f^	2%	-	12%	13%	3%	-	8%	3%
17F^f^	-	-	-	9%	-	-	9%	-
20^f^	-	-	-	1%	1%	1%	1%	-
6C	6%	-	40%	50%	6%	-	6%	19%
7C	-	-	-	-	-	-	-	56%
10B	-	-	-	-	-	-	33%	-
13	-	-	33%	25%	25%	-	25%	-
15A	20%	-	60%	59%	55%	5%	47%	-
16F	-	-	18%	11%	11%	7%	7%	7%
22A	-	-	-	-	-	-	-	50%
23A	-	-	50%	48%	44%	-	48%	8%
23B	-	-	8%	5%	-	-	-	4%
24F	-	-	-	100%	100%	-	50%	-
28A	-	-	-	-	-	33%	33%	-
34	-	-	13%	13%	13%	-	13%	7%
35B	57%	4%	38%	52%	-	-	-	30%
35D	50%	-	-	67%	-	-	-	-
35F	-	-	-	13%	13%	-	7%	-

Abbreviations: AXO, ceftriaxone using the parenteral meningitis interpretive standard; CHL, chloramphenicol; CLA, clarithromycin; CLI, clindamycin; DOX, doxycycline; ERY, erythromycin; PEN, penicillin using the parenteral meningitis Clinical and Laboratory Standards Institute interpretive standard; SXT, trimethoprim/sulfamethoxazole

^a^ “-” denotes no resistance (0%) to the antimicrobial

^b^ Component of PCV13

^c^ Component of PCV15

^d^ Component of PCV20

^e^ Serotypes 15B and 15C were grouped together as 15B/C because of reported reversible switching between them *in vivo* during infection, making it difficult to precisely differentiate between the two types (18,19)

^f^ Component of PPV23

**Table 5:** Percentage of antimicrobial resistance of invasive *Streptococcus pneumoniae* serotypes collected, 2022

Serotype	Percentage of isolates with antimicrobial resistance^a^							
	PEN	AXO	ERY	CLA	CLI	CHL	DOX	SXT
1^b^	-	-	-	-	-	-	33%	33%
3^b^	-	-	4%	4%	1%	6%	6%	1%
4^b^	-	-	9%	9%	7%	3%	14%	12%
14^b^	75%	-	50%	50%	50%	-	50%	75%
7F^b^	-	-	3%	3%	-	-	-	-
9V^b^	69%	3%	72%	71%	-	-	70%	72%
18C^b^	-	-	33%	33%	17%	-	33%	17%
19A^b^	40%	2%	77%	77%	58%	2%	47%	42%
19F^b^	4%	-	4%	4%	4%	-	4%	4%
23F^b^	67%	-	67%	67%	33%	33%	33%	67%
22F^c^	-	-	50%	49%	2%	2%	3%	-
33F^c^	-	-	73%	73%	-	-	-	27%
15B/C^d,e^	3%	-	25%	27%	10%	-	18%	3%
10A^d^	-	-	29%	-	-	-	-	-
11A^d^	3%	-	33%	34%	3%	-	3%	3%
12F^d^	-	-	30%	31%	-	3%	35%	36%
8^d^	-	-	3%	3%	-	-	2%	-
9N^f^	5%	-	5%	5%	-	-	8%	3%
17F^f^	22%	-	11%	11%	-	-	-	-
20^f^	-	-	8%	9%	8%	-	11%	2%
6C	-	-	50%	50%	17%	17%	33%	17%
6D	-	-	-	-	-	100%	100%	100%
7C	-	-	-	-	-	-	8%	69%
13	-	-	40%	40%	40%	-	60%	60%
15A	15%	-	38%	38%	31%	4%	27%	4%
16F	-	-	5%	5%	5%	5%	5%	-
17A	-	-	100%	100%	100%	-	100%	-
23A	-	-	26%	29%	26%	-	29%	9%
23B	-	-	11%	8%	-	-	-	29%
24A	-	-	-	-	-	-	-	100%
24F	-	-	67%	67%	67%	-	67%	-
28A	-	-	-	-	-	50%	50%	-
35B	57%	-	36%	36%	7%	7%	7%	14%
31	-	-	17%	17%	-	-	-	-
34	8%	-	-	-	-	-	-	-
38	-	-	40%	40%	-	-	40%	20%

Abbreviations: AXO, ceftriaxone using the parenteral meningitis interpretive standard; CHL, chloramphenicol; CLA, clarithromycin; CLI, clindamycin; DOX, doxycycline; ERY, erythromycin; PEN, penicillin using the parenteral meningitis Clinical and Laboratory Standards Institute interpretive standard; SXT, trimethoprim/sulfamethoxazole

^a^ “-” denotes no resistance (0%) to the antimicrobial

^b^ Component of PCV13

^c^ Component of PCV15

^d^ Component of PCV20

^e^ Serotypes 15B and 15C were grouped together as 15B/C because of reported reversible switching between them *in vivo* during infection, making it difficult to precisely differentiate between the two types (18,19)

^f^ Component of PPV23

**Figure 4:**
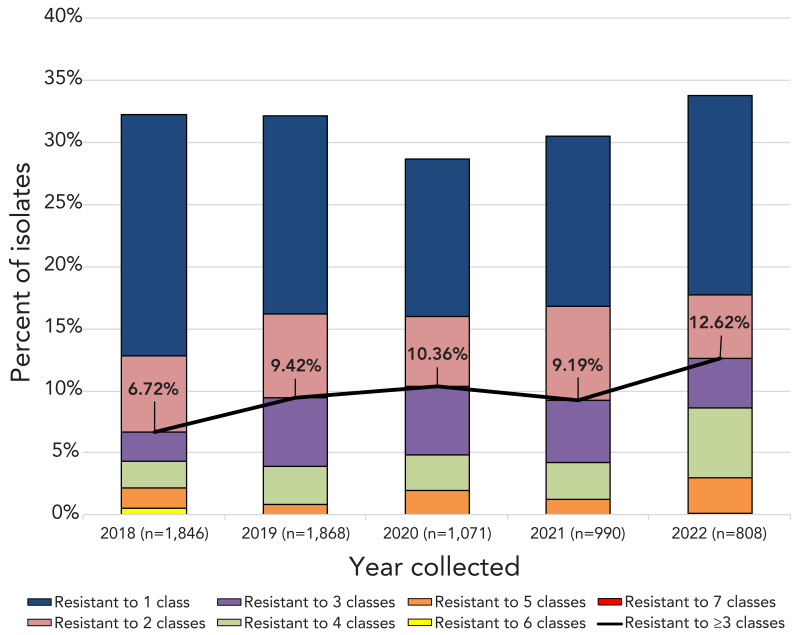
Annual trend of multidrug resistance of invasive *Streptococcus pneumoniae*, 2018–2022^a^ ^a^ Antimicrobial classes include: beta-lactams (amoxicillin/clavulanic acid, penicillin using meningitis breakpoints, ceftriaxone using meningitis breakpoints, imipenem, and meropenem); macrolides (clarithromycin); fluoroquinolones (levofloxacin); tetracyclines (doxycycline); folate pathway inhibitors (trimethoprim-sulfamethoxazole); phenicols (chloramphenicol); lincosamides (clindamycin); and oxazolidinones (linezolid)

Multidrug-resistant IPD increased from 6.7% (n=124) of the isolates tested in 2018 to 12.5% (n=135) in 2022 (*p*<0.0001) (**Figure 4**, Appendix, **Table S9**). Of the serotypes where 10 or more isolates were collected in 2021, the highest rates of MDR were in 15A (50%, n=10), 23A (44%, n=11), 19A (38.7%, n=12) and 9V (28.6%, n=35). In 2022, the highest rates of MDR were identified in 9V (70.7%, n=41), 19A (43.5%, n=27), 15A (30.8%, n=8) and 23A (26.5% n=9) (Table 3, Appendix, **Figure S25a**). The most common MDR pattern in 2021 was macrolide-clindamycin-tetracycline (n=30), including 10 serotype 23A (Appendix, **Table S10a**). For 2022, beta-lactam-macrolide-tetracycline-trimethoprim/sulfamethoxazole was the most common MDR pattern (n=43) with 9V accounting for 41 of these. Serotypes 15A and 23A, for both 2021 and 2022, were resistant to macrolides, clindamycin and tetracycline (n=10 and n=17, respectively). Multidrug resistant serotype 9V isolates were most commonly resistant to four antimicrobial classes (beta-lactam, macrolide, tetracycline and trimethoprim/sulfamethoxazole; n=41), while the most common MDR pattern for serotype 19A was beta-lactam-macrolide-clindamycin-tetracycline-chloramphenicol (n=20) (Appendix, **Figure 25b** and **Table S10b**).

## Discussion

The national incidence rate of IPD in Canada for 2021 was 5.6 cases per 100,000 population, which was very similar to 2020 incidence levels (5.9 cases), but far below the incidence in pre-COVID years that ranged from a low of 9.0 cases in 2009 to a high of 10.9 cases in 2018 (Figure 1). The lower rate can be partially attributed to continued COVID-19 non-pharmaceutical intervention strategies (NPIs) instituted in Canada in 2020, such as masking and physical distancing, working and schooling from home, and travel restrictions (24,25). Global studies of pneumococcal disease and co-infection with viruses, such as respiratory syncytial virus (RSV), influenza and metapneumovirus, suggest that decreased incidence of IPD is not only due to NPIs but also associated with decreased circulation of these viruses during COVID-19 lockdown (26–31). A comprehensive interrupted time series study by Rybak *et al.* that included multiple surveillance systems in France concluded that as pneumococcal carriage rates did not change during periods of NPI use, decreased IPD could be linked to decreased viral infection (29). Gradual lifting of COVID-19 restrictions occurred in Canada in 2022, including a total removal of all travel restrictions in October (24,32). There is concern that a period of increased IPD may occur due to “immunity debt” (lack of stimulation to immune systems) in children, following the lifting of COVID-19-related protective measures (27,33,34). Canadian incidence rates for young children aged <1 year and 1 to 4 years jumped from 5.95 to 10.27 and 6.13 to 9.51 cases per 100,000 population, respectively, from 2020 to 2021. An increase was not seen in older age groups (Figure 1). Although incidence rates for IPD are not yet available for 2022, Canada will likely follow the same trend as other countries. The Invasive Respiratory Infection Surveillance (IRIS) Consortium analyzed surveillance data from over thirty countries, including Canada, and reported a worldwide decrease in IPD incidence during the COVID-19 lockdown followed by an increase late in 2021 (35). Increases in the number of IPD isolates received by NML coincided with the lifting of NPIs, particularly in the last quarter of 2022 (Appendix, Figure S1).

PCV13 serotypes 3 and 4 remained the most common serotypes overall for 2021 and 2022. While the prevalence of serotype 3 saw a dip during the 2021 period of NPI strategies in Canada, serotype 4 continued to rise over this same time. This trend can possibly be attributed to the population dynamics and age groups associated with these serotypes. Multiple studies in the western regions of North America show an association of serotype 4 to adults at risk due to homelessness and drug and alcohol abuse (36–38). Serotype 3 is commonly associated with multiple age groups who would have been more influenced by NPIs than the at-risk populations associated with serotype 4 (39). Poor immunogenicity of serotype 3 remains an issue; preliminary *in vitro* immunogenicity studies of the PCV15 vaccine formulation show increased immune response to serotype 3 in comparison to PCV13, but real-world evidence is needed to corroborate these studies (40–42).

Antimicrobial resistance rates for clarithromycin and erythromycin remained high (both around 24%) but did not trend upward during the study period. Of note is an increase in penicillin resistance (4.7%–8.3%), which can be attributed to an increase in penicillin-resistant serotypes 9V and 19A collected during 2021 and 2022. Over the five-year study period from 2018 to 2022, there was a significant increase in MDR among the isolates tested (6.7%–12.5%, *p*<0.0001). Serotypes 15A and 19A, which have historically exhibited high levels of MDR in Canada, remain a concern; however, similar to the results of the SAVE study described by Adam *et al.*, increased diversity of MDR serotypes was seen (43). Seventy-one percent of all serotype 9V tested exhibited MDR in 2022 as well as 27% of serotype 23A. This will be crucial to monitor going forward, as a steady increase of common MDR serotypes could have a significant impact on patient outcomes in the future.

## Limitations

Caution should be exercised when interpreting the data presented in this report. Provinces and territories may only submit a subset of their isolates to NML for testing. Numbers of isolates submitted to NML versus information submitted to CNDSS may differ due to differences in submission protocols from the provinces. Data for 2020 and 2021 may not be reflective of actual trends, as the COVID-19 pandemic impacted disease incidence in all age groups. Significant increases may have been driven by the large increase in isolates collected in 2022.

## Conclusion

The incidence of IPD in Canada varied very little from 2020 and 2021 after a significant decrease from 2019 to 2020 (incidence rates for 2022 are not available at the time of printing). PCV13 vaccine serotypes 3 and 4 are a major concern in adult age groups, and 15B/C in children <5 years of age. Continued surveillance of IPD serotypes and antimicrobial resistance in Canada is important to monitor existing trends, identify new trends, and assess the effect of newly recommended PCV15 and PCV20 vaccines.

## Appendix

Supplemental figures and tables are available upon request to the author.

Table S1: Annual incidence of invasive pneumococcal disease cases per 100,000 population in Canada by age group, 2010–2021

Figure S1: Number of invasive Streptococcus pneumoniae isolates collected each quarter for <15 years of age and ≥15 years of age, 2018–2022

Figure S2a: Clinical isolation site of invasive pneumococcal disease collected in 2021, by age

Figure S2b: Clinical isolation site of invasive pneumococcal disease collected in 2022, by age

Figure S3a: Percentage of invasive Streptococcus pneumoniae isolates from blood in 2021, by serotype

Figure S3b: Percentage of invasive Streptococcus pneumoniae isolates from blood in 2022, by serotype

Figure S4a: Percentage of invasive Streptococcus pneumoniae isolates from cerebrospinal fluid in 2021, by serotype

Figure S4b: Percentage of invasive Streptococcus pneumoniae isolates from cerebrospinal fluid in 2022, by serotype

Figure S5a: Percentage of invasive Streptococcus pneumoniae isolates from other sterile sites in 2021, by serotype

Figure S5b: Percentage of invasive Streptococcus pneumoniae isolates from other sterile sites in 2022, by serotype

Figure S6a: Prevalence of invasive Streptococcus pneumoniae serotypes isolated in 2021 for the <2, 2–4 and 5–14-year age groups

Figure S6b: Prevalence of invasive Streptococcus pneumoniae serotypes isolated in 2022 for the <2, 2–4, and 5–14-year age groups

Figure S7a: Prevalence of invasive Streptococcus pneumoniae serotypes isolated in 2021 for the 15–49, 50–64 and ≥65-year age groups

Figure S7b: Prevalence of invasive Streptococcus pneumoniae serotypes isolated in 2022 for the 15–49, 50–64 and ≥65-year age groups

Figure S8: Prevalence of invasive Streptococcus pneumoniae serotypes in the <2-year age group, 2018–2022

Figure S9: Prevalence of invasive Streptococcus pneumoniae serotypes in the 2–4-year age group, 2018–2022

Figure S10: Prevalence of invasive Streptococcus pneumoniae serotypes in the 5–14-year age group, 2018–2022

Figure S11: Prevalence of invasive Streptococcus pneumoniae serotypes in the 15–49-year age group, 2018–2022

Figure S12: Prevalence of invasive Streptococcus pneumoniae serotypes in the 50–64-year age group, 2018–2022

Figure S13a: Number of invasive Streptococcus pneumoniae isolates collected in 2021, by region and serotype

Figure S13b: Number of invasive Streptococcus pneumoniae isolates collected in 2022, by region and serotype

Figure S14a: Prevalence of the ten most common invasive Streptococcus pneumoniae serotypes collected in Western Canada, 2021

Figure S14b: Prevalence of the ten most common invasive Streptococcus pneumoniae serotypes collected in Western Canada, 2022

Figure S15a: Prevalence of the ten most common invasive Streptococcus pneumoniae serotypes collected in Central Canada, 2021

Figure S15b: Prevalence of the ten most common invasive Streptococcus pneumoniae serotypes collected in Central Canada, 2022

Figure S16a: Prevalence of the ten most common invasive Streptococcus pneumoniae serotypes collected in Eastern Canada, 2021

Figure S16b: Prevalence of the ten most common invasive Streptococcus pneumoniae serotypes collected in Eastern Canada, 2022

Figure S17a: Prevalence of invasive Streptococcus pneumoniae serotypes collected in Northern Canada, 2021

Figure S17b: Prevalence of invasive Streptococcus pneumoniae serotypes collected in Northern Canada, 2022

Figure S18: Proportion of invasive pneumococcal disease isolates by vaccine for the <2-year age group, 2018–2022

Table S2: Proportion of vaccine serotypes for the <2-year age group, 2018–2022

Figure S19: Proportion of invasive pneumococcal disease isolates by vaccine for the 2–4-year age group, 2018–2022

Table S3: Proportion of vaccine serotypes for the 2–4-year age group, 2018–2022

Figure S20: Proportion of invasive pneumococcal disease isolates by vaccine for the 5–14-year age group, 2018–2022

Table S4: Proportion of vaccine serotypes for the 5–14-year age group, 2018–2022

Figure S21: Proportion of invasive pneumococcal disease isolates by vaccine for the 15–49-year age group, 2018–2022

Table S5: Proportion of vaccine serotypes for the 15–49-year age group, 2018–2022

Figure S22: Proportion of invasive pneumococcal disease isolates by vaccine for the 50–64-year age group, 2018–2022

Table S6: Proportion of vaccine serotypes for the 50–64-year age group, 2018–2022

Table S7: Proportion of vaccine serotypes for the ≥65-year age group, 2018–2022

Figure S23: Proportion of invasive pneumococcal disease isolates by vaccine for all age groups, 2018–2022

Table S8: Proportion of vaccine serotypes for all age groups, 2018–2022

Figure S24: Antimicrobial resistance trends of invasive Streptococcus pneumoniae isolates, 2018–2022

Table S9: Multidrug resistance of invasive Streptococcus pneumoniae isolates, 2018–2022

Figure S25a: Invasive Streptococcus pneumoniae serotypes by resistance to different antimicrobial classes, 2021

Figure S25b: Invasive Streptococcus pneumoniae serotypes by resistance to different antimicrobial classes, 2022

Table S10a: Multidrug resistance profiles of invasive Streptococcus pneumoniae serotypes, 2021

Table S10b: Multidrug resistance profiles of invasive Streptococcus pneumoniae serotypes, 2022

Table S11a: Number of invasive Streptococcus pneumoniae isolates serotyped by the National Microbiology Laboratory (NML) in comparison to the total number of cases reported to Canadian Notifiable Diseases Surveillance System (CNDSS), 2021
